# Risks and Benefits of Green Spaces for Children: A Cross-Sectional Study of Associations with Sedentary Behavior, Obesity, Asthma, and Allergy

**DOI:** 10.1289/ehp.1308038

**Published:** 2014-08-26

**Authors:** Payam Dadvand, Cristina M. Villanueva, Laia Font-Ribera, David Martinez, Xavier Basagaña, Jordina Belmonte, Martine Vrijheid, Regina Gražulevičienė, Manolis Kogevinas, Mark J. Nieuwenhuijsen

**Affiliations:** 1 Centre for Research in Environmental Epidemiology (CREAL), Barcelona, Spain; 2 CIBER Epidemiología y Salud Pública (CIBERESP), Barcelona, Spain; 3 IMIM (Hospital del Mar Medical Research Institute), Barcelona, Spain; 4 Institut de Ciència i Tecnologia Ambientals (ICTA), Universitat Autònoma de Barcelona, Cerdanyola del Vallès, Spain; 5 Departament de Biologia Animal, Biologia Vegetal i Ecologia, Universitat Autònoma de Barcelona, Cerdanyola del Vallès, Spain; 6 Department of Environmental Sciences, Vytauto Didziojo Universitetas, Kaunas, Lithuania

## Abstract

Background: Green spaces have been associated with both health benefits and risks in children; however, available evidence simultaneously investigating these conflicting influences, especially in association with different types of greenness, is scarce.

Objectives: We aimed to simultaneously evaluate health benefits and risks associated with different types of greenness in children, in terms of sedentary behavior (represented by excessive screen time), obesity, current asthma, and allergic rhinoconjunctivitis.

Methods: We conducted a cross-sectional study of a population-based sample of 3,178 schoolchildren (9–12 years old) in Sabadell, Spain, in 2006. Information on outcomes and covariates was obtained by questionnaire. We measured residential surrounding greenness as the average of satellite-derived Normalized Difference Vegetation Index (NDVI) in buffers of 100 m, 250 m, 500 m, and 1,000 m around each home address. Residential proximity to green spaces was defined as living within 300 m of a forest or a park, as separate variables. We used logistic regression models to estimate associations separately for each exposure–outcome pair, adjusted for relevant covariates.

Results: An interquartile range increase in residential surrounding greenness was associated with 11–19% lower relative prevalence of overweight/obesity and excessive screen time, but was not associated with current asthma and allergic rhinoconjunctivitis. Similarly, residential proximity to forests was associated with 39% and 25% lower relative prevalence of excessive screen time and overweight/obesity, respectively, but was not associated with current asthma. In contrast, living close to parks was associated with a 60% higher relative prevalence of current asthma, but had only weak negative associations with obesity/overweight or excessive screen time.

Conclusion: We observed two separable patterns of estimated health benefits and risks associated with different types of greenness.

Citation: Dadvand P, Villanueva CM, Font-Ribera L, Martinez D, Basagaña X, Belmonte J, Vrijheid M, Gražulevičienė R, Kogevinas M, Nieuwenhuijsen MJ. 2014. Risks and benefits of green spaces for children: a cross-sectional study of associations with sedentary behavior, obesity, asthma, and allergy. Environ Health Perspect 122:1329–1335; http://dx.doi.org/10.1289/ehp.1308038

## Introduction

Exposure to greenness has been associated with both health benefits and risks. An emerging body of evidence has linked exposure to greenness with improving both perceived and objective physical and mental health and well-being, probably through increasing physical activity, reducing psychophysiological stress, enhancing social contacts, and reducing noise, heat, and air pollution levels (Bowler et al. 2010; Gill et al. 2007; Lee and Maheswaran 2011; Maas et al. 2009a; Müller-Riemenschneider et al. 2013; Nowak et al. 2006; Pereira et al. 2012, 2013). In children, for example, exposure to greenness has been associated with reduced sedentary behavior and obesity (Bell et al. 2008; Cohen et al. 2006; Epstein et al. 2006; Roemmich et al. 2006; Wolch et al. 2011), which is of public health importance, considering the emerging pandemic of childhood obesity worldwide (Kimm and Obarzanek 2002; Malecka-Tendera and Mazur 2006). However, evidence of a beneficial influence of exposure to greenness on physical activity and obesity has not been consistently reported by all studies (Lovasi et al. 2011; Timperio et al. 2008). Similarly, the available literature on the association of exposure to greenness with allergy and asthma in children is inconsistent. Although some studies have reported positive associations between greenness and allergic conditions or exacerbation of asthma in children in relation to greeness (DellaValle et al. 2012; Lovasi et al. 2013), others have reported no associations or even evidence of protective effects (Hanski et al. 2012; Lovasi et al. 2008; Maas et al. 2009b; Pilat et al. 2012).

These inconsistencies might reflect variation in health influences related to differences in the timing of exposure (e.g., early-life exposure vs. exposure later in life) or the type of greenness (e.g., parks, forests, and residential surrounding greenness) evaluated by different studies. They also might reflect potential conflicting effects of greenness on these health outcomes. For example, greenness can increase the risk of allergic conditions and asthma through production of pollen (DellaValle et al. 2012; Lovasi et al. 2013) and fungal spores (Bartra et al. 2009; De Linares et al. 2010) or through exposure to pesticides/fertilizers (Corsini et al. 2012). On the other hand, green spaces may help reduce the prevalence of these conditions by reducing sedentary behaviors and obesity, and improving air quality (Lovasi et al. 2008; Pilat et al. 2012). Green spaces can also enhance the biodiversity of the living environment at both macrobiota and microbiota scales, which in turn has been inversely associated with dysfunctions of the immune system such as atopy (Hanski et al. 2012; Rook 2013). Few available studies have simultaneously investigated these conflicting influences, particularly in association with different types of greenness. Such studies are needed to provide a more holistic view of the potential harms and benefits of exposure to greenness and help health professionals and policy makers better incorporate the research evidence into recommendations, targeted interventions, policies, and urban planning.

The overarching aim of the present study was to simultaneously investigate the potential health benefits and risks associated with exposure to different types of greenness in children. Toward this aim, we evaluated associations of residential surrounding greenness and proximity to green spaces (separately for parks and forests) and indicators of sedentary behavior, obesity, asthma, and allergy in schoolchildren.

## Methods

### Study Population

This study was carried out in the context of Positive Health Effects of the Natural Outdoor Environment in Typical Populations of Different Regions in Europe (PHENOTYPE) (Nieuwenhuijsen et al. 2014). Our analysis was based on a population-based sample of schoolchildren in the 4th to 6th year of primary schools (9–12 years old) across Sabadell, Spain, in 2006 (Font-Ribera et al. 2009). Sabadell is a city with about 200,000 inhabitants located 20 km northwest of Barcelona in the Catalonia autonomous region, Spain. It has a Mediterranean climate characterized by hot and dry summers, mild winters, and maximum precipitation and vegetation during autumn and spring (Alcaraz-Segura et al. 2009).

To recruit study participants, we contacted 58 primary schools, of which 53 agreed to participate in the study. Data collection was conducted during June 2006. Questionnaires and informed consent forms were provided to the parents of the children. The overall response rate was 58% (3,322 questionnaires) (Font-Ribera et al. 2009).

The study was approved by the ethics committee of the research center, and written informed consent was provided by all participants.

### Questionnaire

Parents completed written questionnaires concerning sociodemographic characteristics, other potential covariates, and the outcomes evaluated for this study (Font-Ribera et al. 2009):

*Respiratory outcome*. Respiratory health status of children was assessed using the validated International Study of Asthma and Allergies in Childhood (ISAAC) questionnaire (Mata Fernández et al. 2005) translated into Spanish and Catalan. We asked about “asthma ever” (“Has your child ever had asthma?” followed by the question “Has it been diagnosed by a physician?”), asthma medication in the preceding 12 months and wheezing in the preceding 12 months. “Current asthma” was defined as asthma ever plus having had wheezing or having used asthma medication in the preceding 12 months (Font-Ribera et al. 2009). We used current asthma as our respiratory outcome.

*Allergic outcome*. We applied the ISAAC questionnaire (“In the past 12 months, has your child had a problem with sneezing, or a runny, or blocked nose accompanied by itchy-watery eyes when she/he did not have a cold or the flu?”) to characterize current allergic rhinoconjunctivitis used as our allergic outcome.

*Sedentary behavior.* Noneducational screen time has been used as an indicator of sedentary behavior in schoolchildren (Chinapaw et al. 2011; Swinburn and Shelly 2008; Tremblay et al. 2011). In our study, parents were asked to report the average noneducational screen time children spent in front of television, computer, and/or video game console separately during working days and weekends. According to the American Academy of Pediatrics recommendation of limiting noneducational screen time to 1–2 hr/day (American Academy of Pediatrics, Committee on Public Education 2001; Council on Communications and Media 2011), we constructed a binary (yes/no) variable (hereafter referred to as “excessive screen time”) indicating whether the child spent > 1 hr during each working day and > 2 hr during each weekend day on watching television, playing video games, and/or working with computer. We used excessive screen time as a surrogate of sedentary behavior (Council on Communications and Media 2011; McCurdy et al. 2010).

*Body anthropometry*. We calculated body mass index (BMI) as weight/height squared. We used World Health Organization reference values to transform BMI into *z*-scores according to age and sex (de Onis et al. 2007). To determine the overweight/obese status, we applied age- and sex-specific BMI cut points recommended by the Childhood Obesity Working Group of the International Obesity Taskforce to define overweight and obesity (Cole et al. 2000). Because of the small number of obese children (*n* = 83), we pooled overweight and obese participants and constructed a binary variable (hereafter referred to as overweight/obesity) to indicate whether the participant was overweight or obese (yes) or not (no). We used BMI *z*-scores (continuous variable) and obesity/overweight (binary variable) as indicators of body anthropometry.

### Exposure to Greenness

We used two measures of exposure to greenness to address different aspects of such an exposure. We used residential surrounding greenness as a surrogate for general outdoor greenness of the living environment of study participants, and used residential proximity to green spaces as a surrogate for the access to green spaces (European Commision, Expert Group on the Urban Environment 2001). We used ESRI ArcGIS Desktop (version 10; ESRI, Redlands, CA, USA) to process the relevant maps.

*Residential surrounding greenness*. To measure surrounding greenness, we used the Normalized Difference Vegetation Index (NDVI) (Bell et al. 2008; Dadvand et al. 2012a, 2012c) derived from the Landsat 4–5 Thematic Mapper (TM) images at 30 m × 30 m resolution (U.S. Geological Survey 2011). NDVI is an indicator of greenness based on land surface reflectance of visible (red) and near-infrared parts of spectrum (Weier and Herring 2011). It ranges between –1 and 1, with higher numbers indicating more greenness. To achieve maximum exposure contrast, we looked for available cloud-free Landsat TM images during springs/autumns (i.e., the maximum vegetation period of the year for our study region) of 2005–2007 (the relevant years to our study period) from the NASA’s Earth Observing System Data and Information System (EOSDIS) website (https://earthdata.nasa.gov/). On the basis of this search we generated our NDVI map using the image obtained on 18 May 2007 (see Supplemental Material, Figure S1).

For each participant, surrounding greenness was abstracted as the average of NDVI in buffers of 100 m (Dadvand et al. 2012a, 2012b, 2012c), 250 m (Dadvand et al. 2012b, 2012c; Lovasi et al. 2013), 500 m (Dadvand et al. 2012b, 2012c; Lovasi et al. 2011; Wolch et al. 2011), and 1,000 m (Bell et al. 2008; Lovasi et al. 2013) around her/his geocoded address of residence. The residential addresses at the time of interview were geocoded to the exact address according to the postal code, street name, and house number.

*Residential proximity to green spaces*. To determine green spaces, we used the Urban Atlas map (2007) detailing land use and land cover for large urban areas (> 100,000 inhabitants) across Europe (European Environment Agency 2007). The Urban Atlas, developed by the European Environment Agency, provides data separately on green urban areas (hereafter referred to as parks) and forests (see Supplemental Material, Figure S2). Based on this distinction, we constructed two binary variables (yes/no) indicating whether the child’s residential address was located within 300 m separately from a park or forest. The 300 m distance was selected according to the European Commission general recommendation for access to green space based on the concept of having a green space within a 15-min walk from home (European Commision, Expert Group on the Urban Environment 2001).

### Regression Analyses

*Main analyses*. We used separate logistic mixed-effects models with a random school effect to estimate associations for each exposure and dichotomous outcome, and used linear mixed-effects models to estimate associations between each exposure and BMI *z*-scores. Random intercepts were used to adjust for potential confounding by unmeasured school characteristics. All analyses were adjusted for indicators of individual level socioeconomic status (SES), including higher educational achievement by either parent (none or primary/secondary/university), and the type of school (public/private). In addition, models were adjusted for area-level SES using quintiles of the Urban Vulnerability index (Ministry of Public Works 2012), a measure of neighborhood SES at the census tract level (median area of 0.08 km^2^ for the study area) based on 21 indicators of urban vulnerability grouped into four themes: sociodemographic vulnerability (5 indicators), socioeconomic vulnerability (6 indicators), housing vulnerability (5 indicators), and subjective perception of vulnerability (5 indicators) (Ministry of Public Works 2012). For this study we used the Urban Vulnerability index based on 2001 Spanish Census (Ministry of Public Works 2012). The analyses for the respiratory and allergic outcomes were further adjusted for child’s sex and age, exposure to environmental tobacco smoke at home (yes/no), having older siblings (yes/no), and parental history of asthma (none/either/both). Having an older sibling has been associated with reduced risk of asthma and rhinitis (Ball et al. 2000; Biagini et al. 2006). We adjusted the analyses of overweight/obesity for sport activity at school or sport facilities (> 6 hr/week, yes/no) (Physical Activity Guidelines Advisory Committee 2008), and having siblings (yes/no). The analyses for sedentary behavior were controlled for child’s sex and age and having siblings. To facilitate the comparison of estimated associations for the surrounding greenness across different buffer sizes, we reported the results for 1 interquartile range (IQR) increase in average NDVI in each buffer size based on all study population. Accordingly, for each subject we divided the average NDVI value in each buffer by the IQR of average NDVI values for all population in that buffer (0.076 for 100-m buffer, 0.105 for 250-m buffer, 0.120 for 500-m buffer, and 0.097 for 1,000-m buffer). Stata statistical software (version 12; StataCorp, College Station, TX, USA) was used to carry out the analyses.

*Sensitivity analyses*. We conducted a variety of sensitivity analyses for each outcome. For the respiratory outcome, we ran additional models with further adjustment for a history of eczema (yes/no) and for breastfeeding history (any/none), and also estimated associations with cases limited to those confirmed by a physician. For the allergic outcome, we also evaluated further adjustment for a history of eczema and for breastfeeding history. For sedentary behavior we evaluated other definitions of excessive screen time (e.g., noneducational screen time > 1 hr/day for both working days and weekends); did analyses excluding participants not able to do physical exercise due to allergy or respiratory health problems (*n* = 3), other health problems (*n* = 2), and other reasons (*n* = 30); and evaluated effect modification by sex. For overweight/obesity, we ran additional models with further adjustment for attending sport classes at school, and models that excluded participants unable to do physical exercise (as defined above).

*Further analyses*. Network buffers. For the main analyses we abstracted residential surrounding greenness using NDVI averages in a number of circular buffers around the home address of study participants. Circular buffers could address a number of our hypothesized mechanisms such as stress restoration through visual access to greenness, reduction in noise and air pollution, and dispersion of pollens; however, for physical activity, network buffers could be more relevant. To explore this, we developed network buffers of 250 m and 500 m around the home address of each study participant and averaged NDVI over these buffers to abstract a new set of residential surrounding greenness. We repeated the main analyses using this alternative set of exposures.

Effect modification by SES. We tested the statistical significance of multiplicative interactions between measures of green exposure (residential surrounding greenness in a 250-m buffer, residential proximity to a park, and residential proximity to a forest) and measures of SES (parental education, type of school, and quartiles of the Urban Vulnerability index) in relation with outcomes using likelihood ratio tests comparing models with and without interaction terms.

For all aforementioned analyses, the statistical significance was considered at *p* < 0.05.

## Results

### Study Population and Questionnaire

We were able to geocode home addresses for 3,178 (95.7%) of 3,322 participants who returned questionnaires. Those participants whose home addresses were not geocoded (*n* = 144) were not statistically significantly different from the rest of participants with regard to the outcomes and covariates (data not shown). Descriptive statistics for the characteristics of study participants and the prevalence of our investigated outcomes are presented in Table 1. Maps of population density and Urban Vulnerability index values at census tracts across Sabadell are presented in Supplemental Material, Figures S3 and S4, respectively.

**Table 1:** Prevalence of outcomes and description of covariates among the study participants, Sabadell, 2006 (*n* = 3,178).

Variable	*n* (%) or median (IQR)^*a*^
Outcomes
Current asthma	138 (4.4%)
Current allergic rhinoconjunctivitis	582 (18.6%)
Excessive screen time^*b*^	893 (28.4%)
Obese/overweight	628 (23.0%)
Exposures
Surrounding greenness (NDVI average)
100-m buffer	0.038 (0.076)
250-m buffer	0.061 (0.105)
500-m buffer	0.095 (0.120)
1,000-m buffer	0.125 (0.097)
Living within 300 m of a park
Yes	1,399 (44.0%)
No	1,779 (56.0%)
Living within 300 m of a forest
Yes	342 (10.8%)
No	2,836 (89.2%)
Covariates
Age (years)	10.9 (1.6)
Missing (*n*)	24
Sex
Female	1,679 (53.0%)
Male	1,491 (47.0%)
Missing (*n*)	8
Siblings (any)
Yes	2,690 (85.3%)
No	462 (14.7%)
Missing (*n*)	26
Sport activity
≥ 6 hr/week	233 (7.3%)
< 6 hr/week	2,945 (92.7%)
School type
Private	1,332 (41.9%)
Public	1,846 (58.1%)
Parental educational achievement
None or primary (≤ 6 schooling years)	623 (20.7%)
Secondary (between 6 and 12 schooling years)	1,380 (45.8%)
University (more than 12 schooling years)	1,012 (33.6%)
Missing (*n*)	163
Parental asthma
None	2,739 (88.0%)
Either	353 (11.4%)
Both	19 (0.6%)
Missing (*n*)	67
Environmental tobacco smoke at home
No	1,963 (62.1%)
Yes	1,200 (37.9%)
Missing (*n*)	15
^***a***^For continuous variables, median (IQR) and for categorical variables count (percentage) of each category has been reported. ^***b***^Defined as spending > 1 hr during each working day and > 2 hr during each weekend day on watching television, playing video games, and/or working with computer.	

Of the 225 children who reported ever having asthma, 209 (92.9%) were diagnosed by physician. Excessive screen time was positively associated with BMI *z*-score [unadjusted regression coefficient of 0.14; 95% confidence interval (CI): 0.05, 0.22], and the relative prevalence of overweight/obesity [unadjusted odds ratio (OR) = 1.32; 95% CI: 1.09, 1.59] and current asthma (unadjusted OR = 1.39; 95% CI: 0.98, 1.98). Furthermore, each 1-unit increase in BMI *z*-score was positively associated with the relative prevalence of current asthma (unadjusted OR = 1.40; 95% CI: 1.15, 1.70) and current allergic rhinoconjunctivitis (unadjusted OR = 1.17; 95% CI: 1.06, 1.28).

### Exposure to Greenness

The median of average NDVI in larger buffers around participants’ home addresses was larger than the median for smaller buffers. The medians (IQRs) of average NDVI values across buffers of 100 m, 250 m, 500 m, and 1,000 m were 0.038 (0.076), 0.061 (0.105), 0.095 (0.120), and 0.125 (0.097), respectively.

With regard to residential proximity to green spaces, 1,399 (44.0%) and 342 (10.8%) participants lived within 300 m of a park or a forest, respectively. The median of average NDVI values in all buffer sizes was higher for participants living within 300 m of a park or forest compared to those living further away (see Supplemental Material, Table S1).

### Regression Analyses

*Main analyses*. Higher residential surrounding greenness in all buffer sizes was statistically significantly associated with lower relative prevalence of overweight/obesity and excessive screen time (Table 2). Consistently in the adjusted models, higher residential surrounding greenness was associated with lower BMI *z*-scores, which was statistically significant for surrounding greenness in 100-m buffer and nearly statistically significant for 250-m buffer (Table 2). On the other hand, the associations between residential surrounding greenness across 100-m and 250-m buffers and current asthma and current allergic rhinoconjunctivitis were nearly null (Table 2). For surrounding greenness in larger buffers of 500 m and 1,000 m, we observed slightly higher relative prevalences of current asthma and current allergic rhinoconjunctivitis, but associations were not statistically significant (Table 2).

**Table 2:** Unadjusted and adjusted ORs (95% CIs) for dichotomous outcomes and regression coefficients (95% CI) for BMI *z*-scores associated with 1 IQR increase^*a*^ in average NDVI across different buffers around participants’ home addresses, Sabadell, 2006 (*n* = 3,178).

Outcome	100-m buffer	250-m buffer	500-m buffer	1,000-m buffer
Current asthma
Unadjusted	1.03 (0.87, 1.22)	1.04 (0.84, 1.29)	1.05 (0.84, 1.33)	1.04 (0.86, 1.27)
Adjusted^*b*^	1.00 (0.82, 1.21)	1.00 (0.78, 1.27)	1.03 (0.79, 1.34)	1.06 (0.85, 1.32)
Current allergic rhinoconjunctivitis
Unadjusted	0.98 (0.89, 1.08)	1.01 (0.90, 1.14)	1.06 (0.93, 1.21)	1.07 (0.96, 1.19)
Adjusted^*b*^	0.97 (0.88, 1.08)	0.98 (0.87, 1.12)	1.03 (0.90, 1.18)	1.05 (0.94, 1.18)
Excessive screen time
Unadjusted	0.86 (0.78, 0.94)**	0.88 (0.78, 0.99)**	0.91 (0.79, 1.03)	0.94 (0.84, 1.05)
Adjusted^*c*^	0.85 (0.77, 0.93)**	0.84 (0.75, 0.94)**	0.85 (0.74, 0.97)**	0.89 (0.79, 1.00)**
Overweight/obesity
Unadjusted	0.87 (0.78, 0.96)**	0.90 (0.79, 1.02)*	0.97 (0.85, 1.11)	1.00 (0.89, 1.12)
Adjusted^*d*^	0.83 (0.75, 0.93)**	0.81 (0.71, 0.92)**	0.83 (0.72, 0.95)**	0.87 (0.78, 0.98)**
BMI *z*-scores
Unadjusted	–0.04 (–0.09, 0.01)*	–0.02 (–0.08, 0.04)	0.03 (–0.04, 0.09)	0.04 (–0.02, 0.10)
Adjusted^*d*^	–0.05 (–0.10, 0.00)**	–0.05 (–0.12, 0.01)*	–0.03 (–0.10, 0.04)	–0.01 (–0.07, 0.05)
^***a***^0.076 for 100-m buffer, 0.105 for 250-m buffer, 0.120 for 500-m buffer, and 0.097 for 1,000-m buffer. ^***b***^Adjusted for child’s sex and age, exposure to environmental tobacco smoke at home, having older siblings, type of school (public vs. private), parental education, and parental history of asthma. ^***c***^Adjusted for child’s sex and age, parental education, type of school, and having siblings. ^***d***^Adjusted for parental education, type of school, sport activity, and having siblings. *p < 0.10. **p < 0.05.				

For residential proximity to a forest, we observed a similar pattern of associations to residential surrounding greenness in that it was associated with a lower relative prevalence of excessive screen time, nearly statistically significant lower relative prevalence of overweight/obesity, and non-statistically significant lower BMI *z*-scores, but it was not associated with current asthma (Table 3). There was a positive association between residential proximity to a forest and current allergic rhinoconjunctivitis which was not statistically significant (Table 3).

**Table 3:** Unadjusted and adjusted ORs (95% CIs) of binary outcomes and regression coefficients (95% CI) for the continuous outcome associated with living within 300 m of parks and forests, Sabadell, 2006 (*n* = 3,178).

Outcome	Parks	Forests
Current asthma
Unadjusted	1.54 (1.10, 2.15)**	1.00 (0.58, 1.74)
Adjusted^*a*^	1.60 (1.09, 2.36)**	1.02 (0.56, 1.87)
Current allergic rhinoconjunctivitis
Unadjusted	1.17 (0.97, 1.41)*	1.27 (0.95, 1.69)
Adjusted^*a*^	1.10 (0.90, 1.35)	1.27 (0.94, 1.70)
Excessive screen time
Unadjusted	1.01 (0.85, 1.21)	0.65 (0.48, 0.89)**
Adjusted^*b*^	0.91 (0.76, 1.09)	0.61 (0.45, 0.83)**
Overweight/obesity
Unadjusted	0.94 (0.77, 1.13)	0.79 (0.58, 1.09)
Adjusted^*c*^	0.90 (0.74, 1.09)	0.75 (0.54, 1.03)*
BMI *z*-scores
Unadjusted	–0.04 (–0.13, 0.06)	–0.03 (–0.19, 0.12)
Adjusted^*c*^	–0.07 (–0.17, 0.03)	–0.06 (–0.21, 0.10)
^***a***^Adjusted for child’s sex and age, exposure to environmental tobacco smoke at home, having older siblings, type of school (public vs. private), parental education, and parental history of asthma. ^***b***^Adjusted for child’s sex and age, parental education, type of school, and having siblings. ^***c***^Adjusted for parental education, type of school, sport activity, and having siblings. *p < 0.10. **p < 0.05.		

Estimates from adjusted models were negative but not statistically significant for living near a park and associations with excessive screen time, overweight/obesity, and BMI *z*-scores (Table 3). On the other hand, living near a park was associated with an OR of 1.60 (95% CI: 1.09, 2.36) for current asthma and an OR of 1.10 (95% CI: 0.90, 1.35) for current allergic rhinoconjunctivitis (Table 3).

*Sensitivity analyses*. The findings for current asthma stayed consistent after further adjustment of analyses for the history of eczema or breastfeeding or after limiting the cases to those confirmed by a physician (*n* = 131) (data not shown). Similarly, the findings for current allergic rhinoconjunctivitis were robust to additional adjustment of analyses for the history of eczema or breastfeeding (data not shown). Our observed associations for excessive screen time did not change notably after using other indicators of excessive screen time or excluding those participants not able to do physical exercise (data not shown). We did not observe any statistically significant interaction of sex and measures of exposure to greenness in association with excessive screen time (data not shown). Further adjustment of overweight/obesity analyses for attending sport classes at school or excluding the participants unable to do physical exercise did not result in a notable change in findings (data not shown).

*Further analyses*. Network buffers. The Spearman’s correlation coefficient between NDVI averages in circular and network buffers was 0.87 for 250-m buffers and 0.89 for 500-m buffers. There was no notable difference between the findings for the residential surrounding greenness based on network buffers (see Supplemental Material, Table S2), and those of main analyses based on circular buffers in terms of direction and strength of the associations.

Effect modification by SES. For the school type (i.e., private vs. public), none of the interaction terms were statistically significant (data not shown). For parental education, only the interaction with residential proximity to forests in relation with sedentary behavior was statistically significant (*p* = 0.02). After stratifying this analysis based on parental education we observed stronger associations for children whose parents had more education. The OR of excessive screen time associated with residential proximity to a forest was 0.91 (0.53, 1.58), 0.63 (0.41, 0.95), and 0.26 (0.11, 0.61), respectively, for children whose parents had primary education, secondary education, and university education, respectively. For Urban Vulnerability index, only the interaction with residential proximity to parks in association with overweight/obesity was statistically significant (*p* = 0.02). After stratifying this analysis based on quintiles of Urban Vulnerability index, we did not observe a clear trend in associations between residential proximity to parks and overweight/obesity across the strata.

## Discussion

This study is one of the first to estimate simultaneously the potential benefits (i.e., reducing sedentary behavior and overweight/obesity) and harms (i.e., increasing risk of asthma and allergy) associated with exposure to greenness in children. To our knowledge, this study is also the first to separate and compare associations of residential proximity to natural and urban green spaces and asthma, allergy, and sedentary behavior. We observed that higher residential surrounding greenness was associated with lower BMI *z*-score and lower relative prevalence of overweight/obesity and excessive screen time, but was not associated with current asthma and current allergic rhinoconjunctivitis. Similarly, residential proximity to a forest was inversely associated with relative prevalence of excessive screen time and overweight/obesity (nearly statistically significant) but not with current asthma. Residential proximity to a forest was associated with an increased risk of current allergic rhinoconjunctivitis which was not statistically significant. On the other hand, living near a park was associated with higher relative prevalence of current asthma, while its associations with BMI *z*-score and relative prevalence of current allergic rhinoconjunctivitis, obesity/overweight, and excessive screen time were generally weak and did not attain statistical significance.

We found negative associations of residential surrounding greenness and proximity to a forest with excessive screen time. These findings are in line with those of previous studies reporting higher physical activity associated with higher neighborhood greenness or better access to green spaces (Cohen et al. 2006; Epstein et al. 2006; Roemmich et al. 2006). Lower screen time has been associated with lower BMI and lower relative prevalence of overweight/obesity (Cecil-Karb and Grogan-Kaylor 2009; Kimbro et al. 2011; McCurdy et al. 2010), which was supported by our data showing positive associations between excessive screen time and BMI *z*-score and overweight/obesity. Consistently, we found that lower BMI *z*-scores and lower relative prevalence of overweight/obesity were associated with residential surrounding greenness and residential proximity to forests—the same measures of exposure to greenness for which we also observed a negative association with excessive screen time. These findings are consistent with those of other studies reporting reduction in BMI *z*-score and overweight/obesity in children in association with higher neighborhood greenness (defined as NDVI average over a buffer of 1,000 m around residential addresses) (Bell et al. 2008) or proximity to green spaces (McCurdy et al. 2010; Wolch et al. 2011).

The available evidence on the association of exposure to greenness and allergy and asthma in children is inconsistent (DellaValle et al. 2012; Hanski et al. 2012; Lovasi et al. 2008, 2013; Pilat et al. 2012). Similarly, we observed different patterns of association for current asthma and current allergic rhinoconjunctivitis in relation with our different measures of exposure to greenness. Although the associations between current asthma and residential surrounding greenness and proximity to a forest were almost null, there was a higher relative prevalence of current asthma associated with living close to a park. Similarly, current allergic rhinoconjunctivitis was not associated with residential surrounding greenness, but it was positively associated with residential proximity to a park or to a forest, although none of the associations attained statistical significance. Sedentary behavior including noneducational screen time (Sherriff et al. 2009) and obesity have been associated with higher relative prevalence of asthma and allergic conditions in children (McCurdy et al. 2010). The negative association of residential surrounding greenness and proximity to forests with excessive screen time and overweight/obesity can therefore partly explain why these exposures were not associated with current asthma and current allergic rhinoconjunctivitis. Differences in estimated associations of asthma with residential proximity to forests (null) and parks (positive) could also be partly explained by a difference between forests and parks in terms of their flora and the allergenicity of their pollens (Cariñanos and Casares-Porcel 2011). For example, compared to forests with their predominantly native flora, parks in Catalonia have mainly “exotic” species such as *Platanus* sp. that are anemophilous (wind-pollinated) trees and have been shown to cause pollen allergies among local people (Belmonte and Vilà 2004; Cariñanos and Casares-Porcel 2011; Gabarra et al. 2002). Another example is *Parietaria,* a weed that thrives abundantly in urban areas of the Mediterranean costal regions. *Parietaria* pollen is known to have strong allergenicity particularly for people 10–30 years of age (Cariñanos and Casares-Porcel 2011; D’Amato et al. 2007). Furthermore, in our setting, forests were mostly located on the edges of the city while parks were located mostly in the middle of the city (see Supplemental Material, Figure S2). This could be suggestive for some potential differences in the living environment (e.g., in terms of degree of urbanity or air pollution levels) experienced by those living next to a forest and those living next to a park that in turn could have contributed to differences in our evaluated outcomes. However, we did not observe any association between ambient levels of air pollutants (nitrogen dioxide and particulate matter ≤ 10 μm in diameter) at home address of participants and current asthma and allergic rhinoconjunctivitis, as described in Supplemental Material (see Supplemental Material, “Air pollution and asthma and allergy”).

Our study faced some limitations. Our cross-sectional study had a limited capability for a causal inference regarding our evaluated associations. For our main analyses, we could not rule out the likelihood of self-selection bias: Families with higher SES may have been more likely to select living in greener neighborhoods, or families of children suffering from asthma/allergy may have been more likely to choose living away from green spaces. Likewise, generalizability of our findings could have been affected by selection bias in that those families who agreed to participate in the study might be different from those who did not participate with respect to a number of characteristics. We did not have data to evaluate such potential differences. However, there was no statistically significant (*p* = 0.87) difference between public and private schools in their response rates, which might suggest that SES was less likely to play a major role in participating in the study.

We used remote sensing–derived NDVI to assess surrounding greenness. Application of this objective measure of greenness enabled us to take account of small-scale green spaces (e.g., home gardens, street trees, and green verges) in a standardized way; however, NDVI does not distinguish between different types of vegetation, which could be relevant to our investigated associations. By using an NDVI map obtained at a single point of time, we effectively assumed that the spatial distribution of NDVI across our study region remained constant over the study period (2006). The findings of our previous study across the same region supports the stability of the NDVI spatial contrast over seasons and years (Dadvand et al. 2012c).

Furthermore, we could not address the impact of the quality of green spaces in our analyses of residential proximity to green spaces. Quality characteristics of green spaces such as aesthetics, biodiversity, walkability, sport/play facilities, safety, and organized social events have been suggested to affect the use of green spaces for physical activity (McCormack et al. 2010). Moreover, we did not have data on use of smartphones by our study participants, which could be relevant to our characterization of sedentary behavior. However, because the data were collected in 2006, we do not expect that a notable portion of our study sample used smartphones. Also, although the validated ISAAC questionnaire was used to identify children with allergic rhinoconjunctivitis, such identification by parents was prone to subjectivity (e.g., attributing common cold to allergic rhinoconjunctivitis or vice versa), and we did not have data on objective tests (e.g., pollen sensitization) to validate parental reports for this outcome. Additionally, we did not have data on severity, frequency, and duration of episodes of allergic rhinoconjunctivitis, which could be of importance in our analysis. Similarly, we did not have data on ethnicity, residential history, and built environment characteristics, such as access to fast food outlets or street connectivity, that could be relevant to our analyses. Moreover, although we adjusted our analyses for indicators of SES at both individual and area levels, we did not have data on other relevant SES indicators such as family income, and residual SES confounding could not be ruled out.

## Conclusion

We observed two separable patterns of associations between our evaluated health outcomes and exposure to different measures of greenness. Residential surrounding greenness was negatively associated with BMI *z*-scores, overweight/obesity, and sedentary behavior, but was not associated with current asthma or allergic rhinoconjunctivitis. Similarly, residential proximity to forests was negatively associated with sedentary behavior, overweight/obesity, and BMI *z*-scores, and was positively associated with current allergic rhinoconjunctivitis but not current asthma. On the other hand, residential proximity to parks was positively associated with current asthma and weakly associated with allergic rhinoconjunctivitis, but was not associated with significantly reduced sedentary behavior or lower overweight/obesity. These observations could indicate critical roles of the use of green spaces and their flora in determining the direction of the equilibrium between health benefits and risks of exposure to greenness; however, because we did not have data on these factors, we could not directly examine their roles. Furthermore, our observed lower relative prevalence of overweight/obesity and sedentary behavior associated with exposure to greenness, if confirmed by future studies, can be of public health importance, considering the current pandemic of childhood obesity and sedentary behavior worldwide and their notable burden due to the wide range of adverse health outcomes associated with these conditions. Further longitudinal studies are warranted to replicate our findings in other settings. We advise future studies to take account of use, quality, and type of plants in green spaces as well as seasonality in vegetation when evaluating health risks and benefits of contact with greenness.

## Supplemental Material

(3 MB) PDF
