# Supplementary material for: Risks and Benefits of Green Spaces for Children: A Cross-Sectional Study of Associations with Sedentary Behavior, Obesity, Asthma, and Allergy
Source: Environ Health Perspect. 2014 Aug 26;122(12):1329–35. doi: 10.1289/ehp.1308038 (PMC4256701; doi:10.1289/ehp.1308038)
Supplement: (3 MB) PDF [file ehp.1308038.s001.508.pdf]

Environmental Health Perspectives doi:10.1289/ehp.1308038

Supplemental Material

Risks and Benefits of Green Spaces for Children: A Cross-Sectional Study of Associations with Sedentary Behavior, Obesity, Asthma, and Allergy

Payam Dadvand, Cristina M. Villanueva, Laia Font-Ribera, David Martinez, Xavier Basagaña, Jordina Belmonte, Martine Vrijheid, Regina Gražulevičienė, Manolis Kogevinas, Mark J. Nieuwenhuijsen.

Table S1. Median (interquartile range) of average NDVI across buffers of 100 m, 250 m, 500 m, and 1000 m around home addresses separately for participants living within and further than 300 m of a park or forest.

Buffer	Parks ≤ 300 m	Parks > 300 m	p-value[a]	Forests ≤ 300 m	Forests > 300 m	p-value[a]
**100 m**	0.061 (0.090)	0.028 (0.057)	< 0.001	0.174 (0.137)	0.033 (0.061)	< 0.001
**250 m**	0.096 (0.090)	0.034 (0.078)	< 0.001	0222 (0.111)	0.050 (0.082)	< 0.001
**500 m**	0.118 (0.075)	0.052 (0.122)	< 0.001	0.259 (0.091)	0.086 (0.104)	< 0.001
**1000 m**	0.145 (0.080)	0.104 (0.114)	< 0.001	0.284 (0.092)	0.114 (0.085)	< 0.001

[a]p-value for Mann–Whitney U test for difference.

Table S2. Adjusted odds ratios (95% confidence intervals (CI)) of binary outcomes and regression coefficients (95% CI) for the continuous outcome associated with one inter-quartile range increase[a] in average Normalized Vegetation Difference Index (NDVI) across network buffers of 250 m and 500 m around participants' home addresses, Sabadell, 2006 (N=3,178).

Outcome	250 m buffer	500 m buffer
**Binary**		
Current asthma[b]	1.01 (0.83, 1.23)	1.02 (0.82, 1.27)
Current allergic rhinoconjunctivitis[b]	0.96 (0.87, 1.07)	1.00 (0.89, 1.12)
Excessive screen time[c]	0.86 (0.77, 0.95)**	0.83 (0.74, 0.94)**
Overweight/obesity[d]	0.82 (0.73, 0.92)**	0.84 (0.75, 0.95)**
**Continuous**		
BMI z-scores[d]	-0.06 (-0.11, -0.01)**	-0.03 (-0.09, 0.02)

[a]0.065 for 250 m buffer and 0.069 for 500 m buffer. [b]Adjusted for child's sex and age, exposure to environmental tobacco smoke at home, having older siblings, type of school (public vs. private), parental education, and parental history of asthma. [c]Adjusted for child's sex and age, parental education, type of school, and having siblings. [d]Adjusted for parental education, type of school, sport activity, and having siblings.

*p-value <0.10. **p-value <0.05.

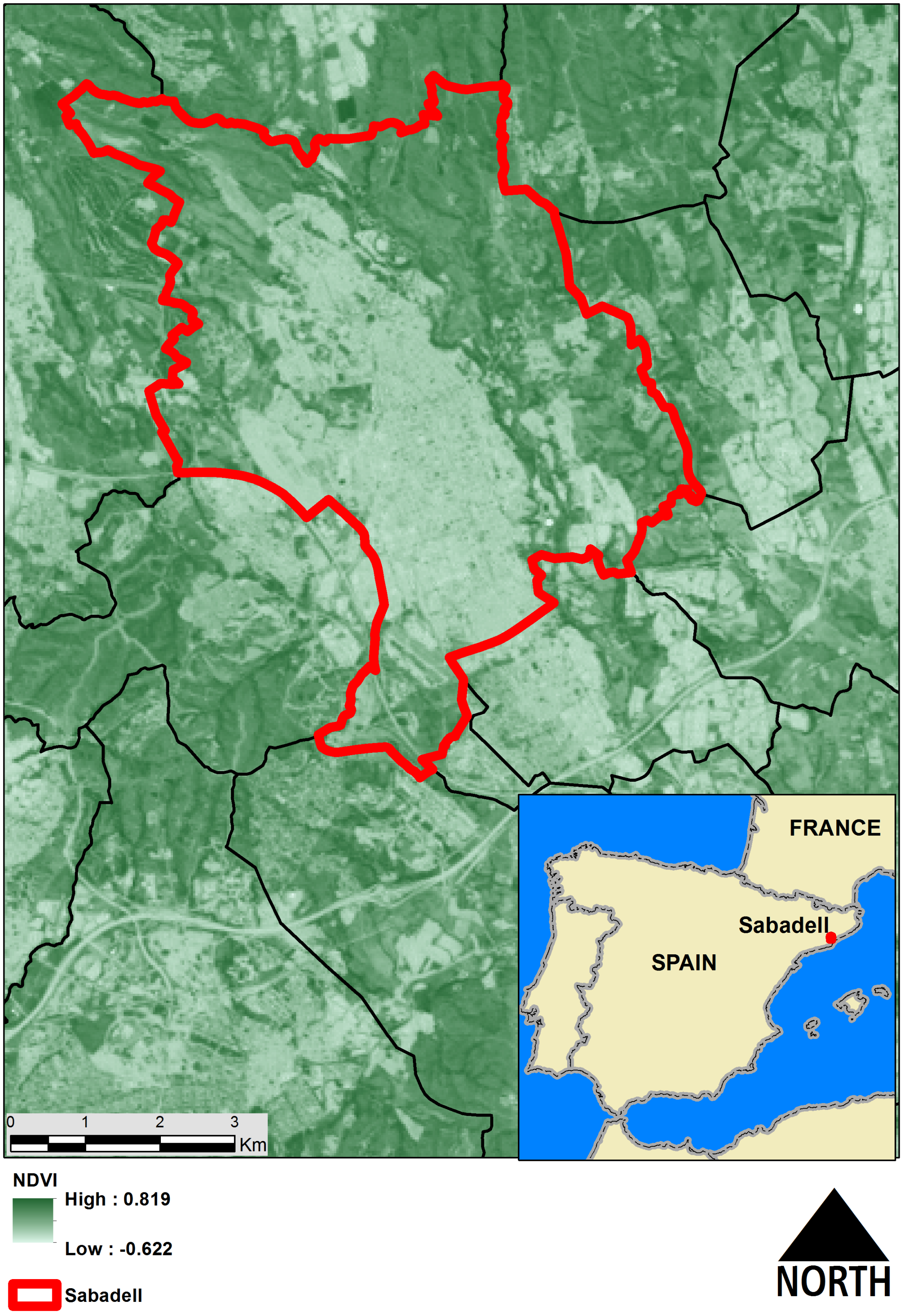

Figure S1. NDVI map of Sabadell, 18th May 2007. Source: Landsat 5 TM.

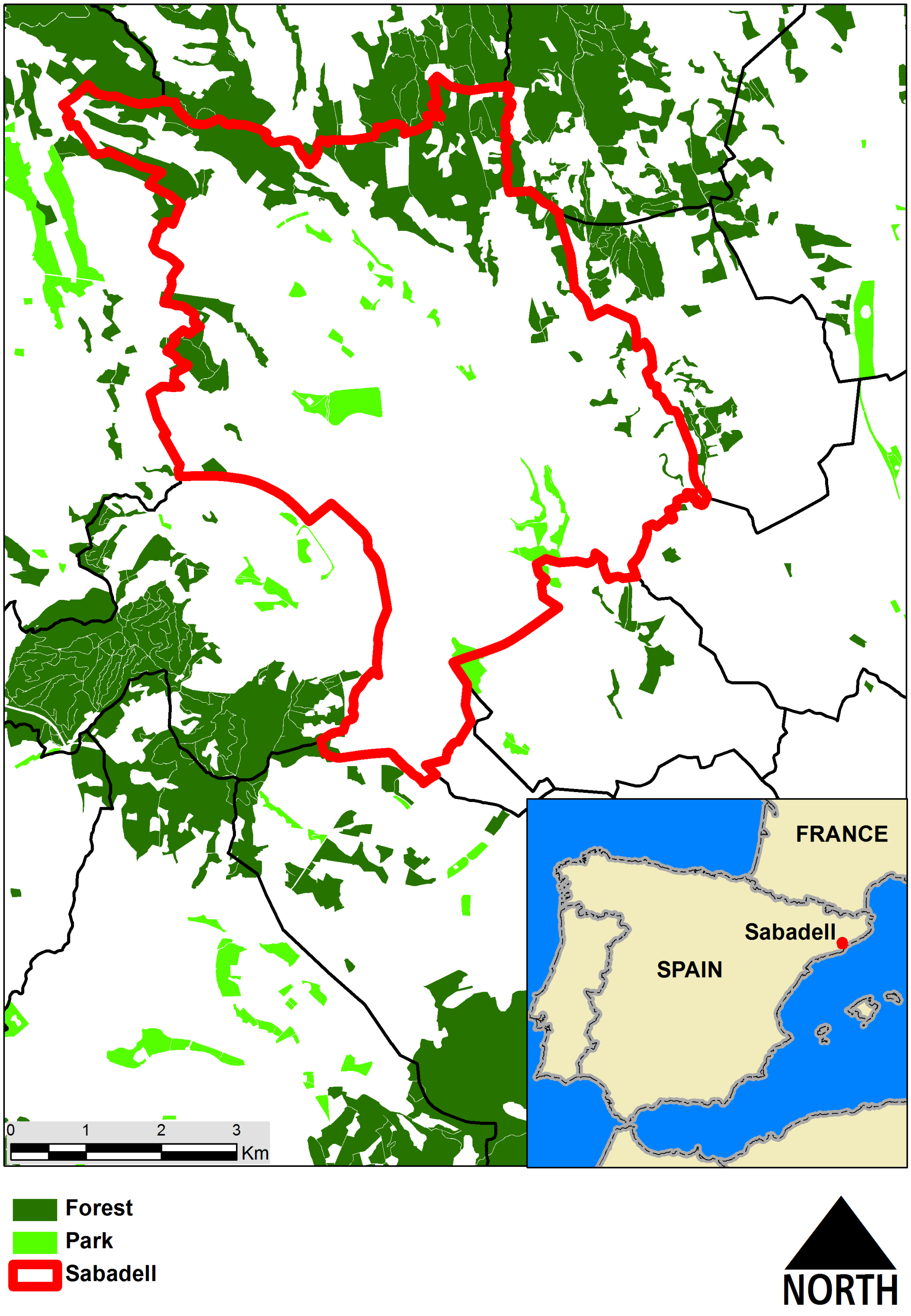

Figure S2. Urban green spaces (Parks) and forests across Sabadell. Source: Urban Atlas (2007).

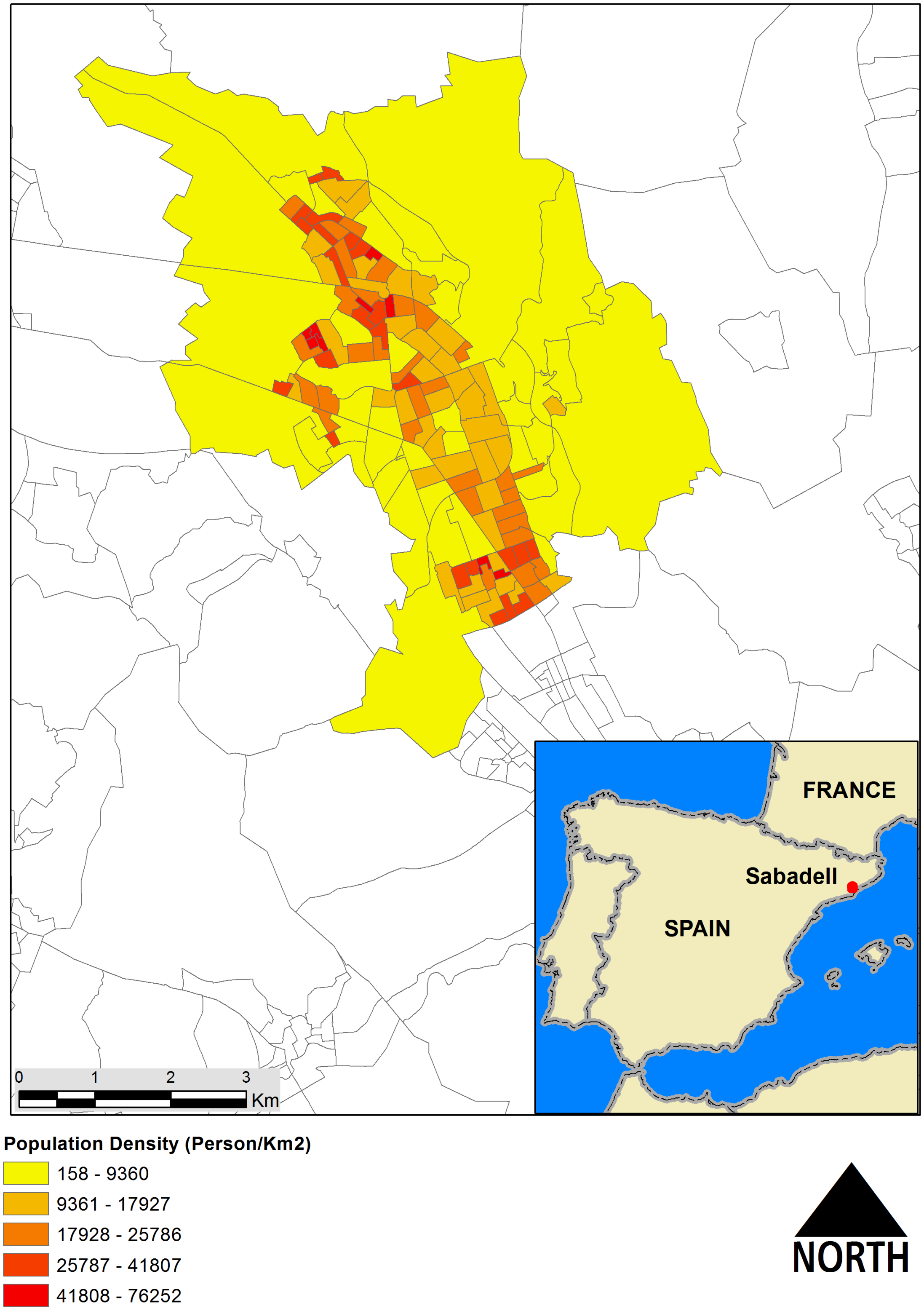

Figure S3. Population density (Person/km^2) at census tracts (Census 2001) across Sabadell.

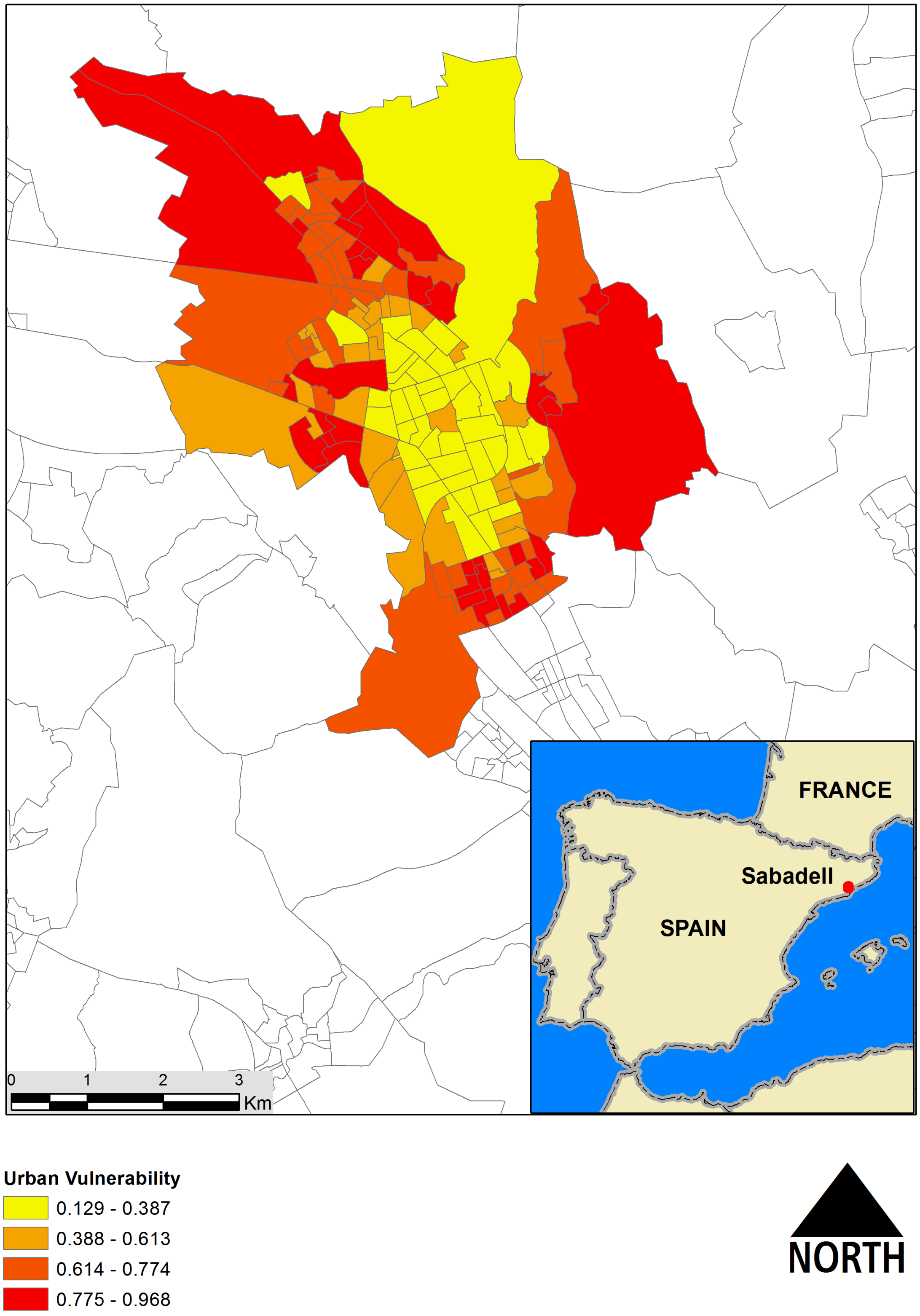

Figure S4. Urban Vulnerability index at census tracts (Census 2001) across Sabadell.

Air pollution and asthma and allergy

We estimated ambient levels of nitrogen dioxide (NO_2) and particulate matter ≤ 10 µm in diameter (PM_{10}) at home address of study participants based on spatial estimates of these pollutants for 2009 generated by land use regression models (Beelen et al. 2013; Eeftens et al. 2012) developed as part of the European Study of Cohorts for Air Pollution Effects (ESCAPE). We evaluated the association between air pollution and current asthma and current allergic rhinoconjunctivitis by replacing indicators of green spaces in adjusted models of *main analyses* by air pollution (one at a time). As presented in the table below, the air pollution was not associated with current asthma and allergic rhinoconjunctivitis.

Table. Adjusted[a] odds ratios (95% CI) of each outcome associated with one unit increase in ambient pollutant levels at participants' home addresses, Sabadell, 2006 (N=3,178).

**Outcome**	**NO_2**	**PM_{10}**
Current asthma	1.00 (0.98, 1.01)	1.02 (0.97, 1.07)
Current allergic rhinoconjunctivitis	1.00 (0.97, 1.03)	1.02 (0.98, 1.06)

[a]Adjusted for child's sex and age, exposure to environmental tobacco smoke at home, having older siblings, type of school, parental education, and parental history of asthma.

References

Beelen R, Hoek G, Vienneau D, Eeftens M, Dimakopoulou K, Pedeli X, et al. 2013. Development of NO2 and NOx land use regression models for estimating air pollution exposure in 36 study areas in Europe – the ESCAPE project. Atmos Environ 72:10-23.

Eeftens M, Beelen R, de Hoogh K, Bellander T, Cesaroni G, Cirach M, et al. 2012. Development of land use regression models for PM2.5, PM2.5 absorbance, PM10 and PMcoarse in 20 European study areas; results of the ESCAPE project. Environ Sci Technol 46(20):11195-11205.
